# Percutaneous transhepatic cholangioscopy combined with endoscopic retrograde cholangiopancreatography for bilateral biliary bridge drainage for malignant biliary obstruction

**DOI:** 10.1055/a-2375-0187

**Published:** 2024-08-08

**Authors:** Gang Tang, Jingyi Zhang, Rui Chen, Jie Zhang, Rongxing Zhou

**Affiliations:** 1 34753 Division of Biliary Tract Surgery, Department of General Surgery, West China Hospital of Sichuan University, Chengdu, China; 2 34753 Department of Medical Ultrasound, West China Hospital of Sichuan University, Chengdu, China

Biliary drainage in advanced malignant hilar biliary obstruction (MHBO) is challenging, especially with Bismuth-Corlette III–IV 1 . By placing a bridge stent between the left and right hepatic ducts, the non-communication system in MHBO can be drained, which is potentially a very promising biliary drainage strategy for MHBO 2 . However, due to the complexity of endoscopic ultrasound-guided hepaticogastrostomy (EUS-HGS) technology, reports on EUS-HGS bridge drainage are still limited. We report a novel approach to bridge bilateral hepatic duct drainage using ultrasound-guided percutaneous transhepatic cholangioscopy (PTCS) combined with endoscopic retrograde cholangiopancreatography (ERCP) in MHBO.

A 57-year-old female with gallbladder cancer presented with jaundice. Computed tomography showed advanced gallbladder cancer with multiple lymph node metastases, hilar bile duct invasion, and biliary obstruction; radical surgery was not possible ( Fig. 1 ). Due to the failure of ERCP, we decided after multidisciplinary discussion to combine ERCP with PTCS for palliative bridging biliary drainage. First, the right hepatic duct was punctured under ultrasound guidance. Then, we dilated the occluded common bile duct with a guidewire and balloon and placed a metal biliary stent. Next, we punctured the left hepatic duct through the right hepatic duct with a puncture instrument under ultrasound guidance, expanded the channel with a balloon, and placed a metal biliary stent to bridge the left and right hepatic ducts. Intraoperative X-ray examination with contrast agent injected through the sinus showed good development of both hepatic ducts, indicating successful bridging, but poor imaging of the distal common bile duct suggested that the distal common bile duct may still be slightly narrow ( Fig. 2 ). Finally, a 10-Fr plastic stent was placed at the distal common bile duct by ERCP ( Video 1 ).

**Fig. 1:**
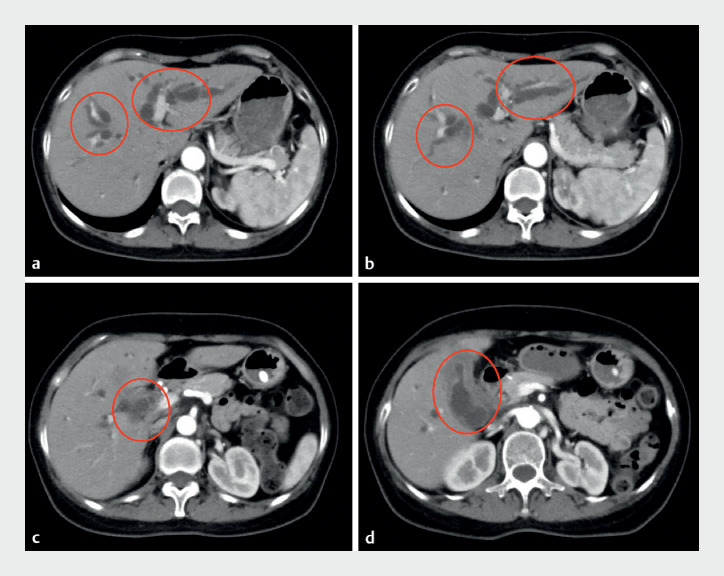
Computed tomography showed advanced gallbladder cancer with multiple lymph node metastases, hilar bile duct invasion, biliary obstruction, and bilateral intrahepatic bile duct dilation. **a–b** The dilated right and left intrahepatic bile ducts. **c–d** Gallbladder cancer with hilar bile duct invasion.

**Fig. 2:**
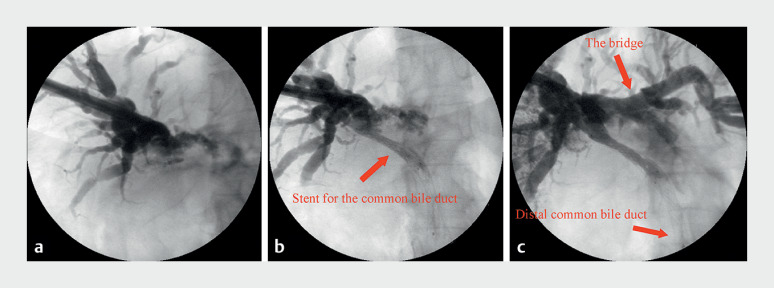
X-ray examination with contrast agent injected through the sinus. **a** The common bile duct before guidewire puncture and balloon dilation. **b** After placing a 10 × 60-mm metal stent for the common bile duct. **c** The left and right intrahepatic bile ducts were bridged with a metal stent (8 × 60 mm); poor imaging of the distal common bile duct.

We successfully performed biliary drainage of the bilateral hepatic duct system using a bridging method from the ultrasound-guided percutaneous transhepatic cholangioscopy combined with endoscopic retrograde cholangiopancreatography in malignant hilar biliary obstruction.Video 1

The jaundice disappeared a few days after surgery. Ultrasound-guided PTCS combined with ERCP for bridging drainage of bilateral hepatic ducts is a feasible treatment option for MHBO.

Endoscopy_UCTN_Code_TTT_1AR_2AJ

## Notes

Endoscopy E-Videos https://eref.thieme.de/e-videos
*E-Videos* is an open access online section of the journal *Endoscopy* , reporting on interesting cases and new techniques in gastroenterological endoscopy. All papers include a high-quality video and are published with a Creative Commons CC-BY license. Endoscopy E-Videos qualify for HINARI discounts and waivers and eligibility is automatically checked during the submission process. We grant 100% waivers to articles whose corresponding authors are based in Group A countries and 50% waivers to those who are based in Group B countries as classified by Research4Life (see: https://www.research4life.org/access/eligibility/ ). This section has its own submission website at https://mc.manuscriptcentral.com/e-videos .

## References

[1] Kongkam P Orprayoon T Boonmee C ERCP plus endoscopic ultrasound-guided biliary drainage versus percutaneous transhepatic biliary drainage for malignant hilar biliary obstruction: a multicenter observational open-label study Endoscopy 2021 53 55 62 10.1055/a-1195-8197 32515005

[2] Pal P Lakhtakia S Endoscopic ultrasound-guided intervention for inaccessible papilla in advanced malignant hilar biliary obstruction Clin Endosc 2023 56 143 154 10.5946/ce.2022.198 36796854 PMC10073857

